# Evaluation of the volume and function of the right atrial appendage using computed tomography

**DOI:** 10.1186/s12872-025-04923-1

**Published:** 2025-07-04

**Authors:** Yang Liu, Tong Pan, Xin Tian, Cai-Ying Li

**Affiliations:** 1 Department of Radiology, The Second Hospital of Hebei Medical University, 215 West Heping Road, Shijiazhuang, Hebei 050011 China; 2 Department of Radiology, Hebei General Hospital, Shijiazhuang, Hebei 050000 China

**Keywords:** Multi-slice spiral CT, Right atrial appendage (RAA), Right atrium (RA), Volume, Function

## Abstract

**Objective:**

To assess the volume and function of the right atrial appendage (RAA) in patients with sinus rhythm using 256-slice spiral computed tomography (CT) angiography.

**Methods:**

A total of 60 patients with normal cardiac CT imaging features were enrolled in this retrospective study. The raw imaging data were used to reconstruct 3D images, which were analyzed to measure the volume of the RAA and right atrium (RA) throughout the cardiac cycle, and to obtain the maximum and minimum volume of the RAA and RA (RAAVmax, RAAVmin and RAVmax, RAVmin). Additionally, the RAA ejection volume (RAAEV), RAA ejection fraction (RAAEF), RA ejection volume (RAEV), and ejection fraction (RAEF) were calculated. Independent sample t-tests were used to analyze the data of the RAA and RA between male and female groups. Pearson correlation analysis was used to analyze the correlation between RAA parameters, RA parameters and basic physiological parameters of the human body.

**Results:**

The volumes of the RAA and RA constantly changed throughout the cardiac cycle, characterized by similar curves and two emptying phases. During 65–85% of the cardiac cycle, the volume of RAA exhibited a minimal fluctuation, indicating a resting state. Male patients had significantly (*P* < 0.05) greater values than female in the RAAVmax, RAAEV, RAVmax, and RAVmin(*P* = 0.003, *P* = 0.001, *P* = 0.031 and *P* = 0.025, respectively). RAAVmax correlated with the patient’s weight, body surface area (BSA), RAVmax, RAVmin and RAAVmin (*r* = 0.434 *P* = 0.001, *r* = 0.363 *P* = 0.004, *r* = 0.331 *P* = 0.010, *r* = 0.352 *P* = 0.006 and *r* = 0.858 *P* = 1.9485E-18, respectively), but did not significantly correlated with height. RAAVmin positively correlated with the weight, BSA, RAVmax, and RAVmin (*r* = 0.434 *P* = 0.011, *r* = 0.363 *P* = 0.035, *r* = 0.331 *P* = 0.006, and *r* = 0.352 *P* = 0.000368, respectively), but did not significantly correlated with height. Additionally, RAAEV correlated with weight, BSA, RAAVmax, and RAAVmin (*r* = 0.426 *P* = 0.001, *r* = 0.356 *P* = 0.005, *r* = 0.885, *P* = 5.9452E-21 and *r* = 0.521 *P* = 0.00020, respectively). RAAEF showed a negative correlation with both RAAVmin and RAVmin (*r*=–0.478 *P* = 0.000112 and *r*=–0.289 *P* = 0.025, respectively).

**Conclusion:**

CT angiography with multi-phase three-dimensional volume reconstruction is very helpful in evaluating the volume and function of RAA, providing an important guidance for diagnosis and treatment of cardiac diseases.

## Introduction

The right atrial appendage (RAA) develops from the primitive atrium and exhibits active systolic and diastolic funtions [1]. Its outer surface is relatively flat, while its inner wall is covered with interlaced pectinate muscles. It is an important site for electrophysiological studies and pacemaker electrode implantation. It is also a target for ablation in some atrial tachycardias [2]. Atrial fibrillation and restrictive cardiomyopathy are significantly risk factors of thrombus formation in the RAA [3, 4]. Studies have shown that compared to the function of left atrial appendage (LAA), RAA function may offer superior predictive value for thromboembolic risk in patients with persistent atrial fibrillation [5].

Transesophageal echocardiography (TEE) remains the gold standard for the study of the morphology, structure, and function of the atrial appendage, as well as for thrombus detection [6]. However, TEE is a semi-invasive procedure that causes pain and discomfort, which limits its widespread clinical application. Magnetic resonance imaging is also used for quantitative research of the atrial appendage. However, it is time-consuming, costly, and often unsuitable for patients with pacemakers [7]. The combination of high-end multi-slice spiral CT(MSCT)and 3D imaging software can intuitively display the morphology and volume of the atrium, ventricle, and atrial appendage [8–10]. Although the anatomical and functional characteristics of the LAA have been extensively studied [11, 12], the RAA remains underexplored, including volume and function.

This study aims to preliminarily evaluate the volume and mechanical function of the RAA in normal individuals using 256iCT, providing valuable information for clinical practice.

## Methods

### Research participants

In this retrospective study, 60 patients with suspected coronary artery disease in the Second Hospital of Hebei Medical University between June 1 and August 31 2020 were enrolled in this study, including 30 males and 30 females with an age range of 35 to 75 (mean, 57.1 ± 10.0) years.All patients had normal sinus rhythm and no evidence of cardiovascular disease on cardiac computed tomography angiography (CCTA) which were performed using retrospective ECG-gating technique. Exclusion criteria included atrial fibrillation, hypertension, coronary artery disease, or other cardiovascular pathologies. Those with poor image quality were excluded from this study.

This study was conducted in accordance with the Declaration of Helsinki (as revised in 2013), and was approved by the Institutional Ethics Committee of The Second Hospital of Hebei Medical University (No. 2018-R245). The institutional review committee waived the requirement for informed consent owing to the retrospective design.

### CT scanning technology

All participants underwent CCTA examination using a Phillips 256-slice spiral CT (Brilliance iCT, Philips Health care, Amsterdam, The Netherlands). The retrospective ECG gating was employed, the parameters were as follows: 80–120 kV,280–350 mAs/rev, collimation 128 × 0.625, pitch 0.18, rotation time, 330 ms, matrix 512 × 512, scanning field of view 250 mm, reconstruction slice thickness 0.9 mm and slice interval 0.45 mm. Patients were coached on breath-hold techniques to minimize motion artifacts. A dual tube high-pressure injector was used to inject non-ionic contrast agent iodixanol (350 mg I/mL) at a flow rate of 4–5 mL/s, with a dose of 0.8 mL/kg. The scan range extended from 0.5 cm below the tracheal bifurcation to the diaphragmatic surface of the heart.

### Image post-processing technology

The raw data were reconstructed into 10 phases (5 to 95%, with an interval of 10%), with a reconstruction slice thickness of 0.9 mm and an interval of 0.45 mm. Definition of the RAA and its base: The superior vena caval orifice was defined as the transitional position between the superior vena cava (SVC) and the RA. The RAA was situated above the superior vena caval orifice on axial images. The Philips EBW 4.6 workstation (Philips Healthcare) was used to obtain the 3D images of the RAA. The base of the RAA, perpendicular to its long axis, is located at the level of the superior vena cava orifice [13].

### Measurement methods

**Fig. 1:**
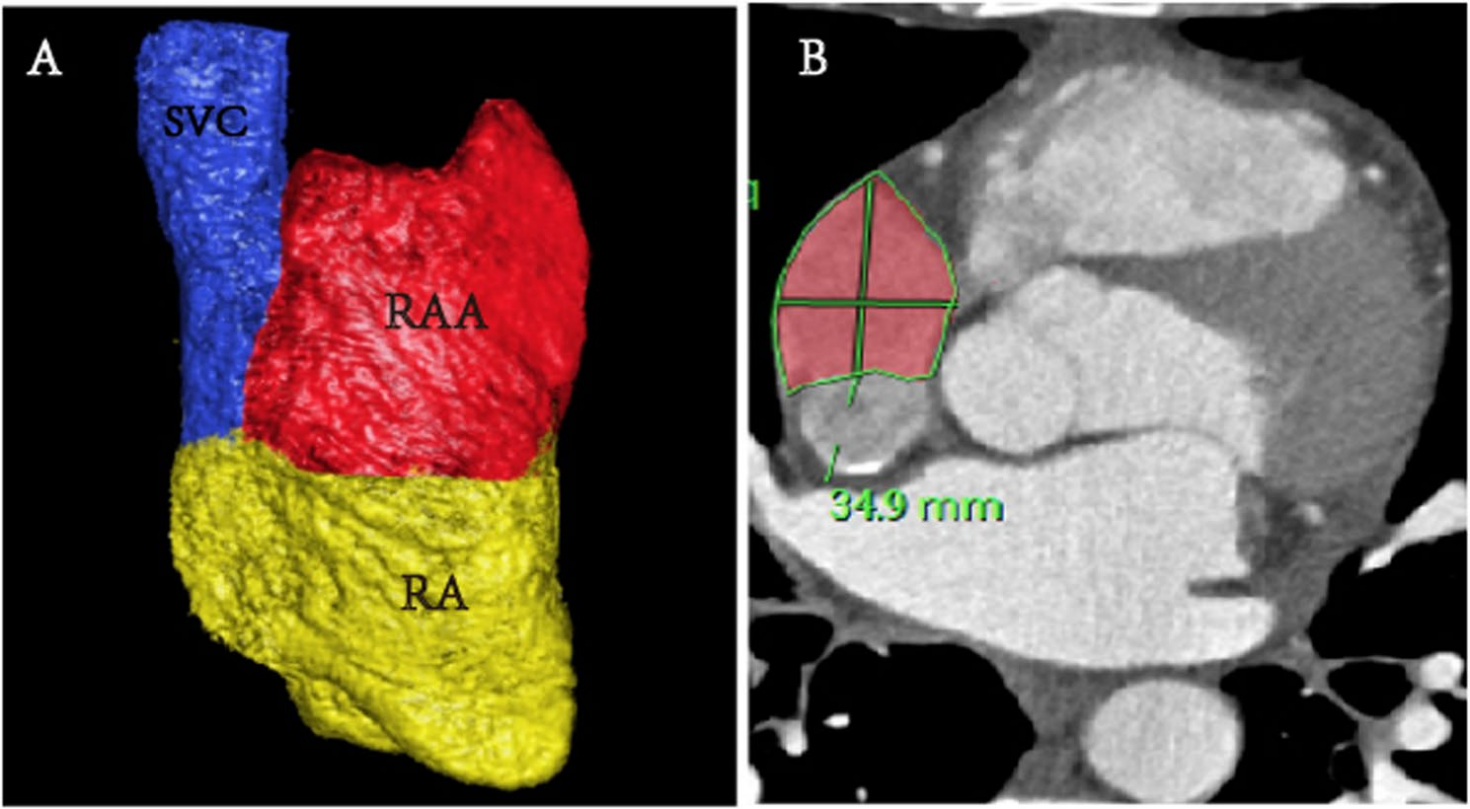
Three-dimensional image of the RAA and RA (**A**). Measurement of the perimeter, and area of the RAA base on axial images (**B**)

**Fig. 2:**
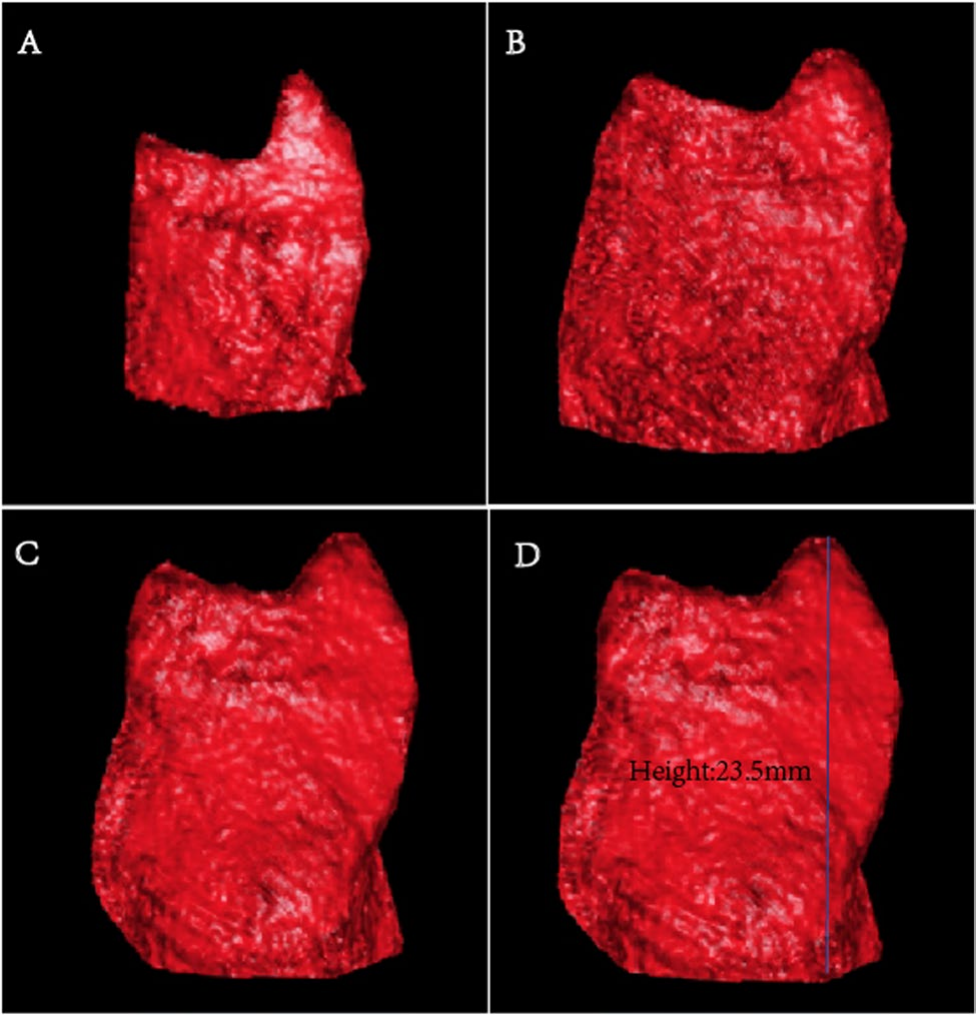
Changes in volume of RAA at different phases (**A**-**C**). the 5% phase (**A**), the 45% phase (**B**), the 75% phase (**C**). RAA height (**D**)

All parameters were analyzed by two experienced radiologists, with over 3 years of experience in cardiac imaging. Consensus in qualitative parameters was reached. Parameters: ①RA and RAA volume: Measured on 3D images of the RAA and RA (Fig. 1A). ② RAA height: The vertical distance from the apex to the base of the RAA was measured (Fig. 2D). ③ Measurement of the long diameter, short diameter, perimeter, and area of the RAA base (Fig. 1B): On the axial image, the base of the RAA was situated at the level of the superior vena cava orifice. The long diameter, short diameter, circumference, and area of the RAA base were all obtained from this image. The above parameters on each phase images were measured to determine the maximal (RAAVmax) and minimal (RAAVmin) volumes of the RAA. Subsequently, the RAA ejection volume (RAAEV) and RAA ejection fraction (RAAEF) were calculated. The parameters of the RA are calculated in a similar way to RAA.

$$\begin{array}{c}\mathrm{RAAEV}\;=\;\mathrm{RAAVmax}-\;\mathrm{RAAVmin}\\\mathrm{RAAEF}\;=\;(\mathrm{RAAVmax}-\;\mathrm{RAAVmin})\;/\;\mathrm{RAAVmax}\times\;100\%\\\end{array}$$

### Statistical analysis

The data were analyzed using the SPSS 25.0 statistical software. The normality test was performed on continuous data using the Shapiro-Wilk test to determine whether the data were considered normally distributed. The data with a normal distribution were represented as mean ± standard deviation (SD), while data with nonnormal distribution were represented as the median (interquartile range). Given that continuous data in this study had followed a normal distribution after normality test, Independent sample t-tests were used to analyze the data of the RAA and RA between male and female groups. Pearson correlation analysis was used to analyze the correlation between RAA parameters, RA parameters and physiological parameters. Paired t-test were used to compare the data of the RAA and RA between the 45% phase and 75% phase. *P* < 0.05 was considered statistically significant.

## Results

### Participants characteristics

In this study, 65 patients with sinus rhythm underwent cardiac CTA examination, 5 patients were excluded due to poor image quality. A total of 60 patients with sinus rhythm were enrolled. Baseline characteristics are listed in Table 1. The height, weight, and BMI in male patients were significantly higher than those in female patients (*P* < 0.05). The female patients demonstrated a higher mean age compared to male patients (*P* < 0.05).

**Table 1:** Clinical characteristics of the male and female

	Male (*n* = 30)	Female (*n* = 30)	*P*
Age (years)	54.3 ± 10.16	59.9 ± 9.25	0.029
Height (mm)	165.77 ± 5.30	158.17 ± 5.59	0.001
Weight (kg)	73.90 ± 10.50	61.93 ± 7.08	0.001
BMI (kg/m²)	26.86 ± 3.31	24.71 ± 2.21	0.004

Data are presented as mean ± SD

*SD* standard deviation

### Volume-time curves of RA and RAA

**Fig. 3:**
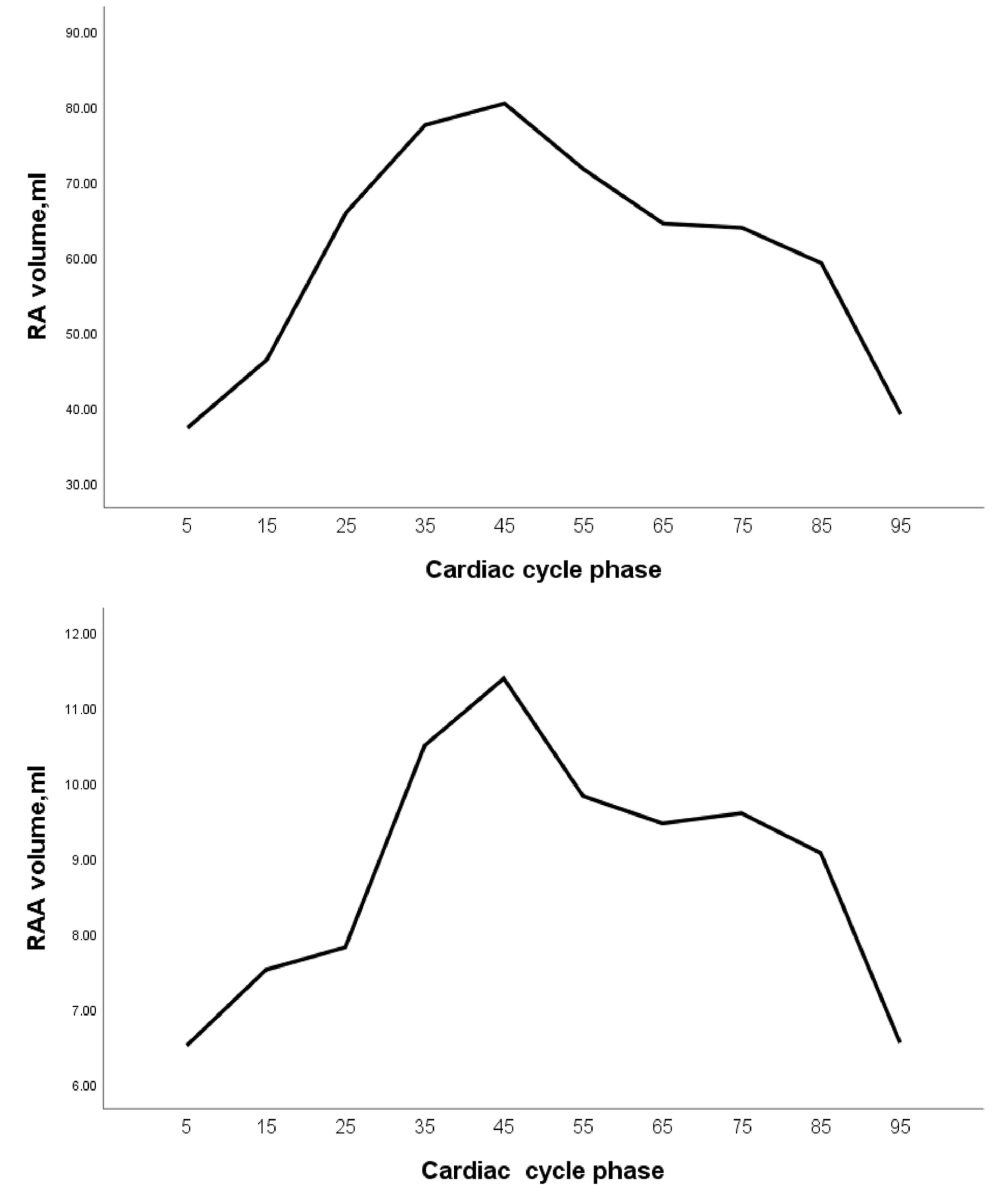
The volume curve of the RA and RAA

The RAA underwent two emptying phases throughout the entire cardiac cycle. The smallest volume of the RAA occurred at the 5% phase (6.51 ± 2.49 mL), followed by a rapid increase reaching its peak at the 45% phase (11.39 ± 3.59 mL). Subsequently, a gradual decline was observed, with a secondary minor peak rising slowly at the 75% phase. From the 65–85% phase, the volume minimally changed, indicating a resting state of the RAA (Figs. 2A and 3). The volume curve of the RA was similar to that of the RAA, undergoing two emptying phases. The volume was lowerst at the 5% phase (37.28 ± 10.11 mL) and reached its peak at the 45% phase (80.44 ± 20.02 mL). The volume curve from 65 to 85% phase was relatively flat, also indicating a resting state (Fig. 3).

### Comparison of the volume and function of the RA and RAA in different sex

The volume and function of the RAA and RA were shown in Table 2. The RAAEF was 53.00 ± 8.77 (%). Male patients had significantly (*P* < 0.05) greater values than female in the the RAAVmax, RAAEV, RAVmax, and RAVmin (*P* = 0.003, *P* = 0.001, *P* = 0.031 and *P* = 0.025, respectively). However, no difference was observed in the RAAVmin, RAAEF, RAEV, and RAEF between males and females(*P* = 0.063, *P* = 0.176, *P* = 0.114, and *P* = 0.411, respectively) (Table 2).

**Table 2:** Comparison of the volume and function of the RA and RAA in different sex

	Male (*n* = 30)	Female (*n* = 30)	t	*P*
RAAVmax (ml)	13.26 ± 3.50	10.68 ± 2.81	3.156	0.003
RAAVmin (ml)	6.06 ± 2.03	5.18 ± 1.57	1.893	0.063
RAAEV (ml)	7.20 ± 2.00	5.50 ± 1.77	3.504	0.001
RAAEF (%)	54.54 ± 7.86	51.46 ± 9.47	1.369	0.176
RAVmax (ml)	87.28 ± 18.29	76.94 ± 17.99	2.206	0.031
RAVmin (ml)	38.61 ± 9.71	32.99 ± 9.18	2.304	0.025
RAEV (ml)	48.66 ± 11.32	43.95 ± 11.44	1.604	0.114
RAEF (%)	55.84 ± 5.91	57.13 ± 6.09	−0.828	0.411

Data are presented as mean ± SD

*SD* standard deviation

### Correlation between RAA parameters, RA parameters and conventional parameters

RAAVmax positively correlated with the patient’s weight, BSA, RAVmax, RAVmin and RAAVmin (*r* = 0.434 *P* = 0.001, *r* = 0.363 *P* = 0.004, *r* = 0.331 *P* = 0.010, *r* = 0.352 *P* = 0.006 and *r* = 0.858 *P* = 1.9485E-18, respectively), but no significant correlation was observed with height. Similarly, RAAVmin was significantly positively correlated with the patient’s weight, BSA, RAVmax, and RAVmin (*r* = 0.434 *P* = 0.011, *r* = 0.363 *P* = 0.035, *r* = 0.331 *P* = 0.006, and *r* = 0.352 *P* = 0.000368, respectively), but was not significantly correlated with height. Additionally, RAAEV aslo exhibited positive correlation with weight, BSA, RAAVmax, and RAAVmin (*r* = 0.426 *P* = 0.001, *r* = 0.356 *P* = 0.005, *r* = 0.885, *P* = 5.9452E-21 and *r* = 0.521 *P* = 0.00020, respectively). RAAEF showed a negative correlation with both RAAVmin and RAVmin (*r*=–0.478 *P* = 0.000112 and *r*=–0.289 *P* = 0.025, respectively) (Table 3).

**Table 3:** The correlation between RAA parameters, RA parameters and physiological parameters

Variables	RAAVmax	RAAVmin	RAAEV	RAAEF
Height	0.030	0.019	0.033	−0.011
Weight	0.434^**^	0.327^*^	0.426^**^	0.097
BSA	0.363^**^	0.272^*^	0.356^**^	0.077
RAAVmax	1	0.858^**^	0.885^**^	0.018
RAAVmin	0.858^**^	1	0.521^**^	−0.478^**^
RAVmax	0.331^**^	0.353^**^	0.230	−0.191
RAVmin	0.352^**^	0.445^**^	0.183	−0.289^*^

All values in this table represent the Pearson correlation coefficients (*r*-values); *P<0.05 **P<0.01

### Comparison of RAA parameters in 45% and 75% phases

As shown in Table 4, except for the depth of the RAA, all other parameters of the RAA in the 45% phase were significantly larger than those in the 75% phase, and the differences were statistically significant (*P* < 0.05).

**Table 4:** Comparison of RAA parameters in different phases

CT-related parameters	45% phases (*n* = 60)	75% phases (*n* = 60)	t	*p*
RAA volume (ml)	11.39 ± 3.59	9.60 ± 3.21	7.597	0.000
RA volume (ml)	80.44 ± 20.02	63.90 ± 11.93	10.850	0.000
RAA depth (mm)	23.58 ± 3.83	23.96 ± 4.18	−0.993	0.325
RAA base				
Long diameter (mm)	37.38 ± 5.52	32.83 ± 3.54	9.184	0.000
Short diameter (mm)	25.57 ± 4.19	24.67 ± 3.68	2.280	0.026
Area (mm²)	772.63 ± 170.73	632.13 ± 117.88	9.518	0.000
Perimeter (mm)	118.14 ± 13.49	104.56 ± 11.12	13.212	0.000

Data are presented as mean ± SD

*SD* standard deviation

## Discussions

The size and function of the RAA are related with various diseases. Atrial fibrillation can increase the volume of the RAA and reduce its function [3]. When mitral stenosis occurs, the systolic function of the LAA and RAA is reduced [14]. In the long-standing persistent atrial fibrillation population, the functional of the LAA and RAA predicted the stages and regional distribution of voltage-defined atrial fibrosis [15]. Decreased ejection fraction of RAA and RA which is caused by atrial fibrillation, leads to blood stasis which promotes thrombosis and atrial remodeling. On the other hand, atrial remodeling can increase the risk of atrial fibrillation [16, 17]. Therefore, further research on the role of the size and function of the RAA is needed.

Previous ultrasound studies had found that the spectral patterns of blood flow in the RAA were relatively similar to those in the LAA, and can be classified into four-phase, three-phase, and two-phase patterns [1, 18]. Our study further explored the function of the RAA, and the volume-time curve of RAA was described for the first time, which was wave-like, dual-phase pattern. This pattern demonstrated that the RAA underwent two processes of blood flow during the entire cardiac cycle: filling, emptying, refilling, and emptying again, with peak volumes at the 45% and 75% phases. This was corresponding to the research results of Bilge et al. [1] about the systolic function of the RAA in normal individuals using TEE. Notably, the volume change curves of the RAA and RA were relatively similar. These findings proved that patients with sinus rhythm experienced synchronous contraction and relaxation of the RAA and RA [1, 19]. The 45% phase and 75% phase represent the two peak filling states of the RAA respectively, but the filling amplitude in the 75% phase was significantly lower compared to the 45% phase. It was found that, except for the height of the RAA, all other parameters of the RAA in the 45% phase were significantly greater than those in the 75% phase. According to the volume-time curve of the RAA, it could be concluded that the fluctuation amplitude of the curve between 65% and 85% phase was relatively small, indicating a minor change in volume, and it was close to the resting state of the RAA. Therefore, measuring parameters such as the volume of the RAA, as well as the diameter and area of RAA base in this phase, were more likely to be accurate.

Previously, we proved that men had greater values than women in the right auricular volume, and height, the base long axis, area and perimeter, and the normal distance [13]. In this study, the RAA volume change in the cardiac cycle was analysed. The RAAVmax, RAAVmin, RAAEF in this study were 6.51 ± 2.49 mL,11.39 ± 3.59mL and 53.00 ± 8.77(%), respectively. The volume range of the RAA was consistent with the results (3.70–17.9 ml) of a study reported by Arisha MJ et al., who measured the volume of RAA in patients without heart disease using echocardiography, and the RAAEF in this study was also similar to the result (54.00 ± 20.56%) reported by Arisha MJ et al. [20]. Male patients had significantly greater values than female in the the RAAVmax, RAAEV, RAVmax, and RAVmin. Both RAAVmax and RAAVmin positivly correlated with body weight, BSA, RAVmax, and RAVmin, but not correlated with the height of RAA. Additionally, RAAEV had a correlation with weight, BSA, RAAVmax, and RAAVmin. In our study, when body weight and BSA increased, the volume and output of the RAA also increased, but There was no significant relationship between the volume and output of the RAA and the individual height. RAAVmax and RAAVmin were positively correlated. RAAEF exhibited a negative correlation with both RAAVmin and RAVmin.

This study had some limitations. This study was retrospective, all patients were examined by routine cardiac CTA with a standard contrast bolus injection protocol. The body weight adapted triphasic contrast media bolus injection protocol [21] was not used to better visualize the structure of the RAA. This study was a single-center, small sample study. The high-speed injection of a contrast agent during MSCT scanning might lead to transient changes in preload hemodynamics, and its impact on measurement results was yet be understood.

## Conclusion

From the 65–85% phase, the volume change of RAA remained relatively minimal, indicating a resting state. Therefore, measuring parameters were more likely to be accurate in this phase. CT angiography with multi-phase three-dimensional volume reconstruction is very helpful in evaluating the volume and function of RAA, providing an important guidance for diagnosis and treatment of cardiac diseases.

## Data Availability

The data that support the findings of this study are available on request from the corresponding author.
